# The economic burden of obesity in Italy: a cost-of-illness study

**DOI:** 10.1007/s10198-021-01358-1

**Published:** 2021-08-04

**Authors:** Margherita d’Errico, Milena Pavlova, Federico Spandonaro

**Affiliations:** 1 Department of Health Services Research, Faculty of Health, Medicine and Life Sciences, CAPHRI, Maastricht University Medical Center, Maastricht University, Universiteitssingel 60, 6229 Maastricht, The Netherlands; 2 Department of Economics and Finance, Faculty of Economics and Finance, University of Rome Tor Vergata, Via Columbia 2, 00133 Rome, Italy; 3 Centre for Economic Applied Research in Health (C.R.E.A. Sanità), Piazza Antonio Mancini 4, 00196 Rome, Italy

**Keywords:** Obesity, Cardiovascular diseases (CVD), Diabetes, Cancer, Cost analysis, Cost-of-illness (COI), A12, B23, C01, H51, I12, I18

## Abstract

**Background:**

Obesity is a complex health disorder that significantly increases the risk of several chronic diseases, and it has been associated with a 5–20-year decrease in life expectancy. The prevalence of obesity is increasing steadily worldwide and Italy follows this trend with an increase of almost 30% in the adult obese population in the last 3 decades. Previous studies estimated that 2–4% of the total health expenditure in Europe is attributed to obesity and it is projected to double by 2050. Currently, there is a lack of sufficient knowledge on the burden of obesity in Italy and most relevant estimates are derived from international studies. The aim of this study is to estimate the direct and indirect costs of obesity in Italy, taking 2020 as the reference year.

**Methods:**

Based on data collected from the literature, a quantitative cost-of-illness (COI) study was performed from a societal perspective focussing on the adult obese population (Body Mass Index (BMI) ≥ 30 kg/m^2^) in Italy.

**Results:**

The study indicated that the total costs attributable to obesity in Italy amounted to €13.34 billion in 2020 (95% credible interval: €8.99 billion < µ < €17.80 billion). Direct costs were €7.89 billion, with cardiovascular diseases (CVDs) having the highest impact on costs (€6.66 billion), followed by diabetes (€0.65 billion), cancer (€0.33 billion), and bariatric surgery (€0.24 billion). Indirect costs amounted to €5.45 billion, with almost equal contribution of absenteeism (€2.62 billion) and presenteeism (€2.83 billion).

**Conclusions:**

Obesity is associated with high direct and indirect costs, and cost-effective prevention programmes are deemed fundamental to contain this public health threat in Italy.

**Supplementary Information:**

The online version contains supplementary material available at 10.1007/s10198-021-01358-1.

## Introduction

Obesity is a multifactorial health disorder characterised by an excessive accumulation of body fat, leading to a significantly increased risk for several chronic diseases, such as diabetes, cardiovascular diseases (CVDs), depression and cancer [1–5]. It has also been associated with a 5–20-year decrease in life expectancy [6, 7]. The prevalence of obesity has been rising worldwide in the past 50 years, to the point of being widely recognised as an “obesity pandemic” [7, 8] with recent estimations of the Global Burden of Disease (GDB) reporting nearly a third of the world population currently classified as overweight or obese [6, 7]. The treatment of obesity and related comorbidities is projected to cost on average 8.4% of the total healthcare expenditure worldwide, with the United States (US) employing nearly 14% of their healthcare budget on obesity and overweight [9]. If the current trend continues unchanged, many European countries are also projected to have an obesity prevalence of 20% or more by 2025, with dramatic consequences on healthcare [10]. Detrimental effects have also been described in the work environment (i.e. sick leave, reduced productivity, and reduced employment), where obesity is projected to cause the loss of the equivalent of 6 million full-time workers by 2050 [9, 11].

In the context of this obesity pandemic, the situation in Italy is not very different. In the past decades, Italy was a symbol of a healthy lifestyle, with an obesity rate of only 8% of the population versus 30% in the US and 21% in the United Kingdom (UK). This rate was significantly lower than that in other Western countries as well, as reported in 2000 by the Organisation for Cooperation and Economic Development (OECD) [12]. However, between 1980 and 2013, the overweight and obesity rate in Italy increased by 27.5% and 47.1% in adults and children, respectively, likely due to lifestyle changes in favour of high-caloric foods and sedentary behaviours [13, 14]. The Italian Government attempted population- and individual-based interventions to contain and reverse this public health issue. For example, the Italian Health Ministry adhered to the Joint Action on Nutrition and Physical Activity (JANPA) initiative promoted by the European Union (EU) as part of a multi-project action aimed to halt the increase in overweight and obesity in children and adolescents by 2020 [15]. In addition, patients with a high body mass index (BMI), BMI ≥ 40 kg/m^2^ or BMI ≥ 35 kg/m^2^ in the presence of other comorbidities, became eligible for weight-loss surgeries fully reimbursed by the Italian National Health System (SSN) [16]. However, the interventions implemented by the Italian government did not seem to result in the desired effects, and latest national reports warn of a constantly increasing trend of obesity in both adults and children [13, 17, 18]. A major factor in this failure is represented by the massive investment directed by the food industry towards advertising food rich in sugar, fats and preservatives that promote obesogenic behaviours across adults and especially youngsters [19]. Moreover, a recent study reported both patients and physicians in Italy having a wrong perception of obesity. While obesity has been officially declared as a chronic progressive disease by the World Obesity Federation [20, 21], only 54% of obese people in Italy seek medical help, and only 36% receive a diagnosis [22].

Accurate measures on the economic burden of obesity are essential for raising awareness around this issue and eventually for developing effective public health interventions to address it. A valid tool to attempt the quantification of these costs is represented by cost-of-illness (COI) studies; economic studies aimed to identify, measure and report in monetary terms all costs that result from a specific disease [23]. COI studies quantify direct costs—directly attributable to patient care such as costs for hospitalisations and drugs, as well as indirect costs, accounting for expenses that do not directly involve patients’ care, but that nevertheless impact society, such as costs for productivity losses or premature mortality [24, 25].

The utilisation of healthcare resources and the resulting attributable costs can be estimated in a COI study by adopting a top–down approach that uses aggregate data, or a bottom–up approach that quantifies the health inputs used to produce specific healthcare services, and subsequently estimates the unit costs [24, 26]. Moreover, based on how epidemiological data are used in the study, COI studies can be divided into prevalence and incidence-based. The prevalence-based approach estimates the total costs of a disease incurred in a given year, and it is the most commonly used. The incidence-based approach implies the calculation of lifetime costs of patients diagnosed in a year of reference, thus providing a baseline against which new interventions can be evaluated [24].

While COI studies are effective tools in assessing the burden of disease, quantifying the accurate direct and indirect costs of obesity remains a complex task due to its multifactorial nature [27, 28]. An Italian COI study used estimates based on prescriptions from general practitioners (GPs) in relation to the BMI of their patients and portrayed a 30%-increase in the healthcare resource utilisation in the Italian obese population [29]. In 2012, a study estimated that only direct medical costs of obesity were responsible for 4% of the total healthcare expenditure in Italy, for a total of €4.5 billion projected to rise in the future [13, 17]. Similarly, a multi-country COI study evaluated the costs of the most common comorbidities of obesity in other European countries (Germany, the Netherlands and the Czech Republic) and estimated a 2–4% total healthcare expenditure attributable to overweight and obesity [30].

Even if the national and international literature already provides information on the magnitude of the obesity burden, the current knowledge does not yet offer an accurate description and projection of the phenomenon for Italian policy-makers [14, 28]. Specifically, most published COI studies on obesity present heterogeneous methodologies and different strategies when accounting for comorbidities or cost categories in their economic analysis, thus hindering the comparability of results across studies [28]. Another major challenge for the evaluation of the burden of obesity in Italy is represented by a marked discrepancy in data of prevalence of BMI classes, especially when comparing databases that collected self-reported data on obesity with databases that collected measured data [14]. For example, according to a report developed by the Italian Institute for Statistics (ISTAT) (multi-purpose analysis) in 2016 on self-reported data, the prevalence of obesity in Italy was 10% [31]. However, a study conducted using an Italian general practice registry, which collected measured data on the distribution of BMI classes, reported a prevalence of obesity of 22% [29].

At present, the national literature on the cost of obesity does not provide sufficiently homogeneous and comparable data to reach a global consensus. Therefore, the present study aims to estimate the economic burden of obesity in Italy, and thus support policy-makers in designing new strategies directed to tackle this constantly growing public health threat.

## Methods

### Study design

A quantitative COI study was conducted to assess the economic burden of obesity in Italy, adopting a societal perspective which considers direct healthcare costs and productivity losses [32]. This study adopted a top–down (population-based) approach, using aggregate data on mortality, morbidity and other disease-related costs and indicators [33]. Costs were calculated using a prevalence-based approach, in which the costs associated with a disease are calculated on an annual basis [34]. After identifying data on the prevalence of obesity and prevalence of obesity-associated comorbidities, costs were estimated using the Population Attributable Fraction (PAF), which represents an estimate of the percentage of the patient population (for each pathology) is attributable to obesity (exposure) and highlights the causal link between the exposure and the attributable pathology [35]. At present, Italy lacks a country-specific check-list for the development and quality assessment of COI studies. Thus, a check-list developed by the “Health Economics” group of the German Network for Healthcare Research has been used as a methodological guideline [36].

### Search of data on obesity prevalence and validation of input data sources

In this study, obesity is defined as having a BMI ≥ 30 kg/m^2^, as indicated by the Centre for Disease Control and Prevention [37]. Data on the prevalence of obesity in Italy was obtained by searching the international and national literature with a snow-ball method starting from two national reports on obesity [17, 38]. The included sources fulfilled the following inclusion criteria: (1) investigation of prevalence of overweight (BMI ≥ 25 to < 30 kg/m^2^) and obesity (BMI ≥ 30 kg/m^2^), (2) inclusion of data on the Italian population, (3) reporting of BMI measurements for male and female adults (≥ 18 years old). Databases that focussed on the prevalence of obesity in children and adolescents were excluded. The extraction of database characteristics was carried out before data quality assessment and included: name of the database, year of publication, sample size, study population age range, adopted definition of adult age, data collection method and BMI classification and data on prevalence (mean value, 95% Confidence Interval). Each measure was double-checked, consulting the original source and extracting values from disaggregate data when possible. If data were provided as stratified by gender, the value was manually derived by calculating the weighted average. Nine criteria based on the methodology developed by the Essnet Validat Foundation [39] were used for the quality assessment of the sources. After extracting the database characteristics, a point was assigned for each of these items: source reliability, data completeness (time frame, geographical area, included variables), inclusion of all BMI classes, reporting the sample size for Italy, clear methodology, consistency of results with other sources, use of the data in previous research, endorsement of a field expert. Two points were assigned if measured data were collected, as previous research highlighted the underestimation of obesity prevalence when collecting self-reported data [40–42]. Supplementary Table 1 reports the original definition of validation and quality criteria for external data sources as described by the Essnet Validat Foundation [39]. The source with the highest score according to the validation tool was chosen for the analysis.

### Bariatric surgery

The weight-loss or bariatric surgery is a primary component in determining costs associated with obesity. According to the guidelines of the Italian Society for Bariatric Surgery (SICOB), patients with a BMI ≥ 40 kg/m^2^ (class III obesity) or a BMI ≥ 35 kg/m^2^ (class II obesity) in the presence of other comorbidities are eligible for a bariatric surgery that is fully paid by the Italian National Health System (SSN) [16]. For this study, the prevalence of class II and class III obesity was retrieved from the literature. However, only 1.4% of eligible patients received on average bariatric surgery in Italy, as reported by the Italian report “Osservatorio PariSanità” [43], which was the value used for our cost calculation.

### Obesity-associated pathologies, relative risks and population attributable fractions

Obesity is a major risk factor for chronic diseases such as metabolic disorders, CVDs, and different types of cancer, which contribute to the overall economic burden of obesity [1–4, 44–46]. In this study, only the most common obesity-associated pathologies were included, namely diabetes and CVDs (angina, atrial fibrillation, cerebrovascular diseases, congestive heart failure, hypertension, ischaemia, myocardial infarction, pulmonary embolism, and stroke). In addition, 11 types of cancer (breast, colon-rectum, kidney, leukaemia, liver, oesophagus, ovaries, pancreas, prostate, thyroid, and uterus) were included, as increasing evidence highlighted the role of obesity in cancer development [6, 47]. They, therefore, represent an interesting case, to date scarcely explored in Italy. Only costs associated with CVDs, diabetes, and cancer were included in the study as they offer an exhaustive measure of the burden of obesity-associated pathologies, with other comorbidities causing negligible costs in comparison [29, 38].

Data on the prevalence of these pathologies were retrieved by conducting a snow-ball literature review in MEDLINE (PubMed) and Embase. Next, the relative risk (RR) attributable to obesity for each pathology was identified. The RR indicates the ratio between the probability of an outcome in the exposed group (with obesity) and the probability of the same outcome in an unexposed group (without obesity) [48]. When data specific for Italy were not available, the RRs were retrieved from studies conducted in countries comparable to Italy in terms of environmental, social, and economic dimensions. The RRs were used to calculate the PAF for each pathology to identify which percentage of the patient population is attributable to obesity (exposure) [35]. The PAF indicates a causal relationship between the exposure and pathology, indicating in this study, not only the population affected by both obesity and another pathology simultaneously but also the one that developed the pathology exclusively because of obesity. The PAF for an obesity-associated pathology *i* was calculated as follows:

$$PAF_{i} = \frac{{P_{o} \left( {RR_{i} - 1} \right)}}{{1 + P_{o } \left( {RR_{i} - 1} \right)}}$$

where *P*_o_ denotes obesity prevalence and *RR*_*i*_ denotes relative risk of the obesity-associated pathology *i.*

The fraction of the population *PC*_O_ with a pathology *i* due to obesity was calculated multiplying the total population affected by that pathology *PC*_*i*_ by the PAF of that pathology *PAF*_*i*_ as follows:

$$PC_{{\text{o}}} = PC_{i} \times PAF_{i}$$

### Cost evaluation

#### Direct costs

The cost evaluation considered both direct costs solely attributable to obesity (i.e. bariatric surgery) and direct costs attributable to obesity-associated pathologies. Cost categories were searched considering all possible direct costs for obesity and obesity-attributable pathologies (i.e. drugs, nursing services, and physician visits). Based on the results of the literature search, only the items relevant to obesity were included in the cost calculation, namely costs for drugs, hospitalisations, monitoring and adverse events. More specifically, hospitalisation costs included costs for primary care, inpatient care, and outpatient care.

The total costs of bariatric surgery were estimated using the most recent Italian estimates from the Italian report “Osservatorio PariSanità”, that reported pro-capita average costs of bariatric surgery in Italy, taking in account acute care, long-term care and rehabilitation [43]. Costs pro-capita were multiplied by the number of class II and class III obese patients, that are eligible for the surgery according to the SICOB guidelines [16]. The Italian rate of patients receiving the surgery on the total eligible population, namely 1.4% [43], was considered for the cost calculation.

The costs attributable to each obesity-associated pathology were estimated by identifying the pro-capita costs in the literature. The *PC*_O_ was multiplied by the costs pro-capita *DC*_*i*_ to obtain total healthcare costs *TC*_*i*_ per year for each pathology as follows:

$$TC_{i} = PC_{{\text{o}}} \times DC_{i}$$

In addition, the MEDLINE (PubMed) and Embase databases were searched using a snow-ball method to obtain data on costs of weight-loss programmes, psychological counselling, and special transportation (i.e. bariatric ambulance).

When needed, costs have been adjusted to 2020, using the CCEMG-EEPI-Centre cost converter, a web-based tool recommended by the World Health Organisation (WHO) to adjust an estimate of costs to a target currency and/or price year [49]. To minimise the risk of double counting when extracting cost items from the literature, the lowest cost per person in the absence of other comorbidities was included. Alternatively, cost categories were filtered out to select the items relevant to each obesity-associated pathology.

#### Indirect costs

The MEDLINE (PubMed) and Embase databases were searched using a snow-ball method to obtain information on productivity loss due to obesity. The cost items specific for Italy available in the literature, namely costs for presenteeism and absenteeism, were included in the calculation of obesity-attributable costs. The average costs pro-capita were extracted from the study by Gupta et al. [50] that adopted the human capital approach to estimate the productivity losses. After identifying the average cost per person, the costs reported for normal-weight individuals were subtracted from those reported for the obese population to obtain obesity-attributable costs. The cost difference was multiplied by the number of obese people to obtain costs at a population level. Costs were adjusted to 2020 using the CCEMG-EEPI-Centre cost converter.

### Sensitivity analyses

A deterministic (one-way) sensitivity analysis was conducted to address the uncertainty of the data included in the model and to validate the study results. The minimum and maximum values of all the variables included in the model were defined by increasing or decreasing each item value by 10%. Seven variables were tested in the deterministic sensitivity analysis, namely obesity prevalence, rate of eligible patients receiving a bariatric surgery, total costs of bariatric surgery, total costs of obesity-associated CVDs, total costs of obesity-associated diabetes, total costs of obesity-associated cancer, total costs of obesity-associated productivity losses. In particular, the minimum and maximum rates of patients that received the bariatric surgery were set at 1% and 3%, respectively, with the purpose of simulating a similar trend to the ones previously reported for other countries [51, 52].

In addition, a probabilistic sensitivity analysis (PSA) for the base case was performed, adopting the Monte Carlo method (second order) and replicating the calculation of the total obesity costs with 1,000 simulations. The estimated values for the PSA were based on the assumed value distribution, point estimates and standard error (SE).

## Results

### Data on prevalence of obesity and validation of input sources

As mentioned above, a purposive literature review was conducted to identify data on prevalence of obesity in Italy. Data on prevalence were extracted from the most recent population-based databases that investigated obesity in the Italian adult population and summarised in Table 1. In particular, the search identified seven national and international databases, whose main characteristics, namely source and year of data publication, data collection (measured or self-reported), sample size and BMI classification definition are reported in Supplementary Table 2. While the age range of the study populations differed markedly across databases (Supplementary Table 2, Age Range), the definition of BMI classes was overall consistent and included four main categories: underweight, normal-weight, overweight and obese. However, the Health Search database further stratified obese patients in class I (BMI ≥ 30 to < 35 kg/m^2^), class II (BMI ≥ 35 to < 40 kg/m^2^) and class III (BMI ≥ 40 kg/m^2^) [53]. If prevalence measures were only provided as stratified by gender, the data were manually derived, calculating the weighted average (Table 1, data marked with an asterisk (^a^)). The complete prevalence data, including details on less recent reports, confidence intervals (CI 95%), gender of the participants and public accessibility, were reported in Supplementary Table 3.

**Table 1:** Prevalence data for body mass index (BMI) classes (obesity, overweight, normal, and underweight) for adults in Italy from seven different databases

Database source and year	Data collection	Underweight BMI < 18.50 (%)	Normal 18.50 ≥ BMI > 25 (%)	Overweight 25 ≥ BMI > 30 (%)	Obese BMI ≥ 30 (%)
Health Search, 2010 [29]	Measured	2.66	40.8	36.88	19.66^a^
Health Search, 2012 [53]	Measured	2.3	38.1	37.4	22.2^b^
Progetto CUORE, 2012 [18, 44]	Measured	–	–	40.5^a^	25.8^a^
Global Burden of Disease, 2013 [6]	Mixed	–	–	49.9^a^	18.2^a^
Global Burden of Disease, 2015 [64]	Mixed	–	–	34^a^	11^a^
EUROSTAT (EHIS), 2015 [54]	Self-reported	3.3	51.9	34.1	10.8
Global Health Observatory, 2016 [92]	Measured	0.8	35.1^a^	41.2^a^	22.9
ISTAT multipurpose, 2018 [93]	Self-reported	3	50.26	35.76	10.98
Indagine Passi, 2018 [94]	Self-reported	3.1^a^	54.4^a^	31.60	10.9

^a^Derived value

^b^Further stratified in Obesity Class I: 72.8%; Class II: 20.1%; Class III: 7.2%

The seven databases presented similar values for the prevalence of underweight individuals in the Italian population (~ 2.6%). On the contrary, prevalence of overweight and obesity differed markedly, with obesity ranging from 10.8% as reported by EUROSTAT [54] to 25.8% as reported by Progetto CUORE (OEC/HES) [18]. Similar values were displayed by databases that collected self-reported (~ 10.5%) or measured data (~ 20%). The GBD study performed in 2015 reported similar values to the ones by ISTAT, EUROSTAT, and PASSI, albeit using both self-reported and measured data.

Due to the heterogeneity of these sources, an input data validation was performed to choose the source to be included in the analysis. Nine validation criteria were adapted from the methodology proposed by the Essnet Validat Foundation [39] (see “Methods” section). The validation matrix and the original criteria formulation are reported, respectively, in Supplementary Tables 1 and 4. The Health Search database was the only source that obtained a full score and its most recent measures (2012) were therefore selected for the analysis (see Supplementary Table 5). The Health Search measures on prevalence of obesity, stratified according to age group and BMI index, are reported in Table 2.

**Table 2:** Prevalence of obesity in Italy stratified by age and BMI classification, adapted from Colao et al. [53]

Prevalence	Normal weight 18.50 ≥ BMI > 25, (%)	Overweight 25 ≥ BMI > 30, (%)	Obese BMI ≥ 30, (%)	Total (%)
18–29 years	62.0*	22.3*	15.8*	100
30–64 years	40.8**	36.4**	22.8**	100
65 + years	32.1	44.2	23.7	100

^*^p < 0.0001 vs 30–64 years and +65 years; **p < 0.0001 vs +65 years

### Bariatric surgery

The number of patients receiving a bariatric surgery was calculated using the prevalence of BMI classes from Colao et al. [53]. As data on prevalence referred to 2012, the Italian population in 2012 was taken from the ISTAT database (ISTAT 2012). The SICOB guidelines consider eligible to bariatric surgery only patients with a BMI ≥ 40 kg/m^2^ or BMI ≥ 35 kg/m^2^ in the presence of other comorbidities [16]. However, only 1.4% of eligible patients on average receive bariatric surgery, as reported by a recent national report [43]. Therefore, this share of the population was used for the cost analysis, resulting in the estimation of 41,880 bariatric surgery in 2020 in Italy, considering a prevalence of obesity of 22.2%.

### Obesity-associated pathologies, relative risks and population attributable fractions

Key obesity-associated pathologies were included in the analysis, namely diabetes, CVDs and cancer (see “Methods”). Diabetes and CVDs were included as their impact on the overall burden of obesity has been widely described in the literature [17, 29, 55]. Moreover, 11 types of cancer were selected and included in the analysis, as increasing evidence described their connection with obesity [56–59] and thus represented a case of specific interest. Only the cancer types whose risk has been associated with obesity were included.

To estimate the cases of diabetes and CVDs attributable to obesity (Table 3), it was necessary to first calculate the number of total cases in Italy. Figures on the prevalence of diabetes and CVDs were obtained from Atella et al. [60], Giampaoli et al. [44] and Moretti et al. [61] (Supplementary Table 6) and multiplied by the Italian adult population of the corresponding year. All data on prevalence referred to 2014, except for cerebrovascular diseases (prevalence in 2012) and pulmonary embolism (prevalence in 2007). Therefore, the Italian population in 2014, 2012 and 2007 was taken from the ISTAT database (ISTAT 2007, 2012, 2014).

**Table 3:** Obesity-associated cases of cardiovascular diseases (CVDs) and diabetes in Italy estimated using the Population Attributable Fraction (PAF)

Disease	Population^a^	Prevalence (%)	Cases in Italy	RR^b^	PAF (%)	Cases due to obesity
Cardiovascular diseases						
Angina	43,054,180	0.8	344,433	1.96^W^	18	60,510
Atrial fibrillation	43,054,180	2.9	1,248,571	1.49^Wa^	10	122,495
Cerebrovascular disease	38,056,749	0.7	266,397	1.54^W^	11	28,517
Congestive heart failure	43,054,180	1.3	559,704	1.79^G^	15	83,514
Hypertension	43,054,180	30.0	12,916,254	2.41^GBD^	24	3,079,196
Ischaemic heart disease	43,054,180	3.5	1,506,896	2^D^	18	273,757
Myocardial infarction	43,054,180	1.0	430,542	1.44^W^	9	38,313
Pulmonary embolism	36,548,850	0.02	6908	3.51^G^	36	2472
Stroke	43,054,180	4.9	2,109,655	1.56^D^	11	233,272
Endocrinological diseases						
Diabetes	43,054,180	8	3,444,334	6.25^D^	54	1,853,785

The PAF was previously calculated for each included pathology, using the relative risks (RR) that were extracted from four different studies

^a^Italian Population > 35, ISTAT 2007, 2012, 2014

^b^RR is derived by calculating the mean value of the female and male RRs retrieved from previously published studies; *GBD*  Global Burden of Disease, 2015; *D*  DYNAMO-HIA, 2010; *G*  Guh et al. 2009; *W*  Wilson et al., 2002; *Wa*  Wanahita et al., 2008

Next, it was necessary to identify the RRs that in the case of obesity indicate the risk for an obese person to develop a certain pathology compared to a normal-weight person. The RRs were extracted from five studies, selecting when possible measured data from European study populations: the DYNAMO-HIA Project, a European report, which includes a systematic review and meta-analysis of RR linked to obesity [62]; the systematic review and meta-analysis of Guh et al. [47] of US (55%) and European studies (40%); the cohort study by Wilson et al., [46] conducted in the US with about 5200 participants; the meta-analysis of Wanahita et al. [63]; and the Global Burden of Disease, which investigated the burden of obesity worldwide [64].

After identifying the RRs for each considered pathology, the PAF was calculated as described in the methods, to estimate which fraction of the population affected by a pathology is attributable to obesity. The calculations performed to obtain the PAF for each obesity-associated pathology are reported in detail in Supplementary Table 7. A similar methodology was employed for estimating the obesity-associated cases of cancer reported in Table 4. However, data on prevalence of all the cancer types included in the study were not available in the literature. Therefore, the proportion of each cancer type from the total of cancer cases diagnosed in Italy in 2014 was obtained from the Associazione Italiana di Oncologia Medica (AIOM) report “I numeri del cancro in Italia, 2014” [65] and multiplied by the total of cancer cases in 2014 to obtain the number of cases specific for each cancer type. Obesity-attributable cancer cases were then estimated using the PAF, as described for the other pathologies.

**Table 4:** Obesity-associated cases of cancer in Italy estimated using the Population Attributable Fraction (PAF)

Cancer type	Proportion (%)^a^	Cases in Italy	RR^b^	PAF (%)	Cases due to obesity
All	100	2,243,953	**–**	**–**	**–**
Breast	23.3	522,235	1.25^D^	5	27,460
Colon-rectum	13.2	296,687	1.25^D^	5	15,600
Kidney	3.8	84,413	1.68^D^	13	11,072
Leukaemia	2.3	51,378	1.11^GBD^	2	1225
Liver	1.0	21,416	1.24^GBD^	5	1083
Oesophagus	0.2	3700	2.3^D^	22	829
Ovaries	1.7	37,829	1.04^GBD^	1	333
Pancreas	0.4	9636	1.08^GBD^	2	168
Prostate	9.7	216,716	1.05^G^	1	2379
Thyroid	3.6	81,129	1.18^GBD^	4	3117
Uterus	4.1	91,689	1.61^GBD^	12	10,936

The PAF was previously calculated for each included pathology, using the relative risks (RR) that were extracted from four different studies

^a^Proportions are taken from the AIOM report, 2014

^b^RR is derived by calculating the mean value of the female and male RRs retrieved from previously published studies; *GBD *Global Burden of Disease, 2015; *D*  DYNAMO-HIA, 2010; *G * Guh et al. 2009

### Cost evaluation

#### Direct costs

This analysis included obesity-attributable direct costs for drugs, hospitalisations, monitoring and adverse events. All costs referred to the year 2020; where needed costs have been adjusted using the CCEMG-EEPI-Centre cost converter (see “Methods” section) [49].

The costs of bariatric surgery were calculated using the costs pro-capita from the Italian report “Osservatorio PariSanità”, that reported an expense of €5779 per patient (Table 5) [43]. To obtain the total costs of bariatric surgery, costs pro-capita were multiplied by the number of surgeries estimated in the year 2020, based on the requirements of SICOB guidelines [16] and the rate of patients receiving bariatric surgery in Italy [43] (Table 6).

**Table 5:** Yearly average direct costs pro-capita for patients receiving a bariatric surgery or for patients affected by diabetes, cancer or cardiovascular diseases (CVDs), adjusted to 2020

Disease	Direct costs pro-capita	Reference
Bariatric surgery	€5779	D’Angela et al. [43]
Diabetes	€353	Marcellusi et al. [66]
Cancer	€4489	Jönsson et al. [73]
Cardiovascular diseases		
Angina^a^	€6066	–
Atrial fibrillation	€3597	Ringborg et al. [67]
Cerebrovascular disease^a^	€6066	–
Congestive heart failure	€12,028	Corrao et al. [68]
Hypertension	€272	Scholze et al. [69]
Ischaemia^a^	€6066	–
Myocardial infarction	€11,584	Mantovani et al. [70]
Pulmonary embolism	€1496	Gussoni et al. [71]
Stroke	€7419	Gerzeli et al. [72]

Costs pro-capita for angina, cerebrovascular diseases and ischaemia were not available in the literature and were, therefore, estimated as average of the other identified CVD costs

^a^Average of the other CVD costs

**Table 6:** Obesity-attributable direct costs in millions of euros for bariatric surgery, cancer, cardiovascular diseases (CVDs) and diabetes, adjusted to 2020

Disease	Direct health costs due to obesity (mln)
Surgical procedures	
Bariatric surgery	€242.0
Neoplasms of	
Breast	€123.3
Colon-rectum	€70.0
Kidney	€49.7
Leukaemia	€5.5
Liver	€4.9
Oesophagus	€3.7
Ovaries	€1.5
Pancreas	€0.8
Prostate	€10.7
Thyroid	€14.0
Uterus	€49.1
Cardiovascular diseases	
Angina	€367.1
Atrial fibrillation	€440.6
Cerebrovascular disease	€173.0
(Congestive) heart failure	€1004.5
Hypertension	€837.5
Ischaemia	€1660.6
Myocardial infarction	€443.8
Pulmonary embolism	€3.7
Stroke	€1730.6
Endocrinological diseases	
Diabetes	€654.4
Total direct health costs	€7891.0

*Mln* millions

Patient costs pro-capita in Italy for the included obesity-associated pathologies were taken from published studies and summarised in Table 5. When possible, the lowest cost pro-capita estimated in the absence of other comorbidities was taken to minimise the double count of pathology-attributable costs. However, the PAF used to estimate the obesity-attributable cases for each pathology implies a causal relationship exposure-outcome, thus already indicating the presence of more pathologies (obesity plus another one). Taking costs in the absence of comorbidities, and thus of non-“comorbid” patients, may result in an underestimation of costs.

Considering costs adjusted to 2020, Marcellusi et al. [66] reported a cost pro-capita of €353 for diabetes without comorbidities. Yearly direct costs were €3597 for atrial fibrillation [67], €12,028 for (congestive) heart failure [68], €272 for hypertension [69], €11,584 for myocardial infarction [70], €1496 for pulmonary embolism [71], and €7419 for stroke [72]. Costs pro-capita for angina, cerebrovascular diseases and ischaemia were not available in the literature. Therefore, the average cost of the six CVDs was calculated and used for further analysis. Similarly, costs specific for the included cancer types could not be found in the literature and were estimated using data from Jönsson et al. [73], which reported total direct costs in Italy for the year 2014. The total costs were divided by the number of cancer patients in Italy in 2014, assuming an equal average pro-capita cost for all cancer patients to obtain costs pro-capita. The number of obesity-attributable cases for diabetes, CVDs and cancer (Tables 3, 4) was multiplied by the costs pro-capita reported in Table 5, to obtain the total obesity-attributable direct costs for each included pathology (Table 6).

Healthcare costs for GP contacts attributable to obesity were excluded, as the studies that reported the volume utilisation for GP visits did not differentiate between visits attributable to obesity alone and to obesity-associated pathologies [74, 75]. Likewise, costs for weight-loss programmes, psychological support and special transportation (i.e. bariatric ambulance) due to obesity were excluded from this study, due to limited data availability in the literature.

#### Indirect costs

Obesity-attributable indirect costs include productivity losses—i.e. costs for presenteeism and absenteeism—and have been adjusted to 2020 using the CCEMG-EEPI-Centre cost converter [49]. Costs pro-capita were taken from Gupta et al. [50], the only available study that provided costs for Italy. Their study included the EU5 (France, Germany, Italy, Spain and the UK) and estimated annual indirect costs using the human capital method, multiplying wages by the percentage of work productivity impairment [50]. Gupta et al. provided estimates as averages of the values obtained for the five included countries (€3409 for normal-weight people; €3633 for class I obese, €4077 for class II obese, €5307 for class III obese) and address the heterogeneity across different population groups by exclusively considering costs for employable people. For the calculations here reported, obesity-attributable costs were obtained by subtracting the costs reported for normal-weight individuals from those reported for the obese.

Absenteeism and presenteeism costs were calculated separately and stratified for the three obesity classes (€129, €415, €857 due to absenteeism in class I, II, III, respectively; €124, €372, €1,299 for presenteeism in class I, II, III, respectively). The number of obese employees was calculated using data on obesity prevalence in 2012 [53] on a total of 10,957,666 individuals (Italian adults in 2012, ISTAT). Costs pro-capita were multiplied by the number of obese employees, resulting in total indirect costs of €2.62 billion for absenteeism and €2.83 billion for presenteeism (Table 7).

**Table 7:** Indirect costs (IC) due to obesity-attributable absenteeism (A) and presenteeism (P)

BMI Class	Prevalence (%)	Obese by BMI class	IC A	IC P	IC A due to obesity	IC P due to obesity	IC A/year (mln) due to obesity	IC P/year (mln) due to obesity
Normal weight (BMI < 25)	–	–	€955	€2709	–	–	–	–
Obese I (BMI 30–35)	72.8	7,977,181	€1084	€2833	€129	€124	€1029	€986
Obese II (BMI 35–40)	20.1	2,202,491	€1369	€3081	€415	€372	€913	€819
Obese III (40 ≥ BMI > 70)	7.2	788,952	€1812	€4008	€857	€1,299	€676	€1025
All classes	100	10,957,666	–	–	–	–	€2618	€2830

Data on prevalence of obesity (for 2012) were taken from Colao et al., and the number of obese was calculated on a total of 10,957,666 individuals (Italian adults in 2012). Costs pro-capita were taken from Gupta et al. and total IC adjusted to the year 2020

*Mln* millions

#### Overall costs

The overall costs attributable to obesity in 2020 for Italy are reported in Table 8 and amounted to €13.34 billion, with €0.24 billion for bariatric surgery, €0.65 billion for diabetes, €6.66 billion for CVDs, €0.33 billion for cancer and €5.45 billion losses of productivity. Direct healthcare costs represented 59.2% of the overall costs, while indirect costs accounted for the 40.8%.

**Table 8:** Costs attributable to obesity in billions of EUR in Italy in 2020

Direct healthcare costs		
Bariatric surgery	€0.24	1.8%
Diabetes	€0.65	4.9%
Cardiovascular diseases	€6.66	49.9%
Cancer	€0.33	2.5%
Total direct costs	€7.89	59.2%
Indirect costs		
Absenteeism	€2.62	19.6%
Presenteeism	€2.83	21.2%
Total indirect costs	€5.45	40.8%
Total	€13.34	100.0%

### Sensitivity analysis

**Fig. 1:**
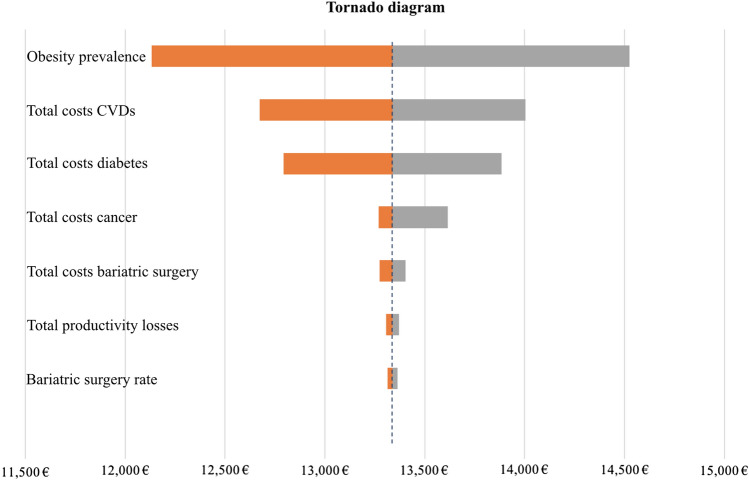
Deterministic (one-way) sensitivity analysis and tornado diagram. Seven variables were tested to address uncertainty of the following parameter values when estimating the economic burden of obesity: (1) obesity prevalence, (2) total costs of bariatric surgery, (3) total costs of obesity-attributable CVDs, (4) total costs of obesity-attributable diabetes, (5) total costs of obesity-attributable cancer, (6) total costs of obesity-associated productivity losses, and (7) rate of eligible patients receiving bariatric surgery. Parameter values are changed through upper and lower bounds to estimate minimum and maximum total obesity costs

The model results were validated by performing a deterministic (one-way) sensitivity analysis that tested seven variables: (1) obesity prevalence, (2) total costs of bariatric surgery, (3) total costs of obesity-attributable CVDs, (4) total costs of obesity-attributable diabetes, (5) total costs of obesity-attributable cancer, (6) total costs of obesity-associated productivity losses, and (7) rate of eligible patients receiving bariatric surgery. Results indicated the minimum and maximum total obesity costs when one of the above variables (parameters) changed through a predefined minimum–maximum bond. The results are reported in a tornado diagram that displays the variation in the overall costs of obesity when varying each of the above variables (parameters) chosen for the deterministic sensitivity analysis (Fig. 1).

The prevalence of obesity was the variable that showed the relatively highest variation in total obesity cost, resulting in a minimum of €12.13 billion when reducing the obesity prevalence by 10% and a maximum of €14.52 billion when increasing it by 10%. Total indirect costs and bariatric surgery rate had a relatively minor impact on total obesity cost, determining a variation of plus/minus €33.3 and €24.20 million, respectively, when taking their minimum or the maximum value in this study.

**Fig. 2:**
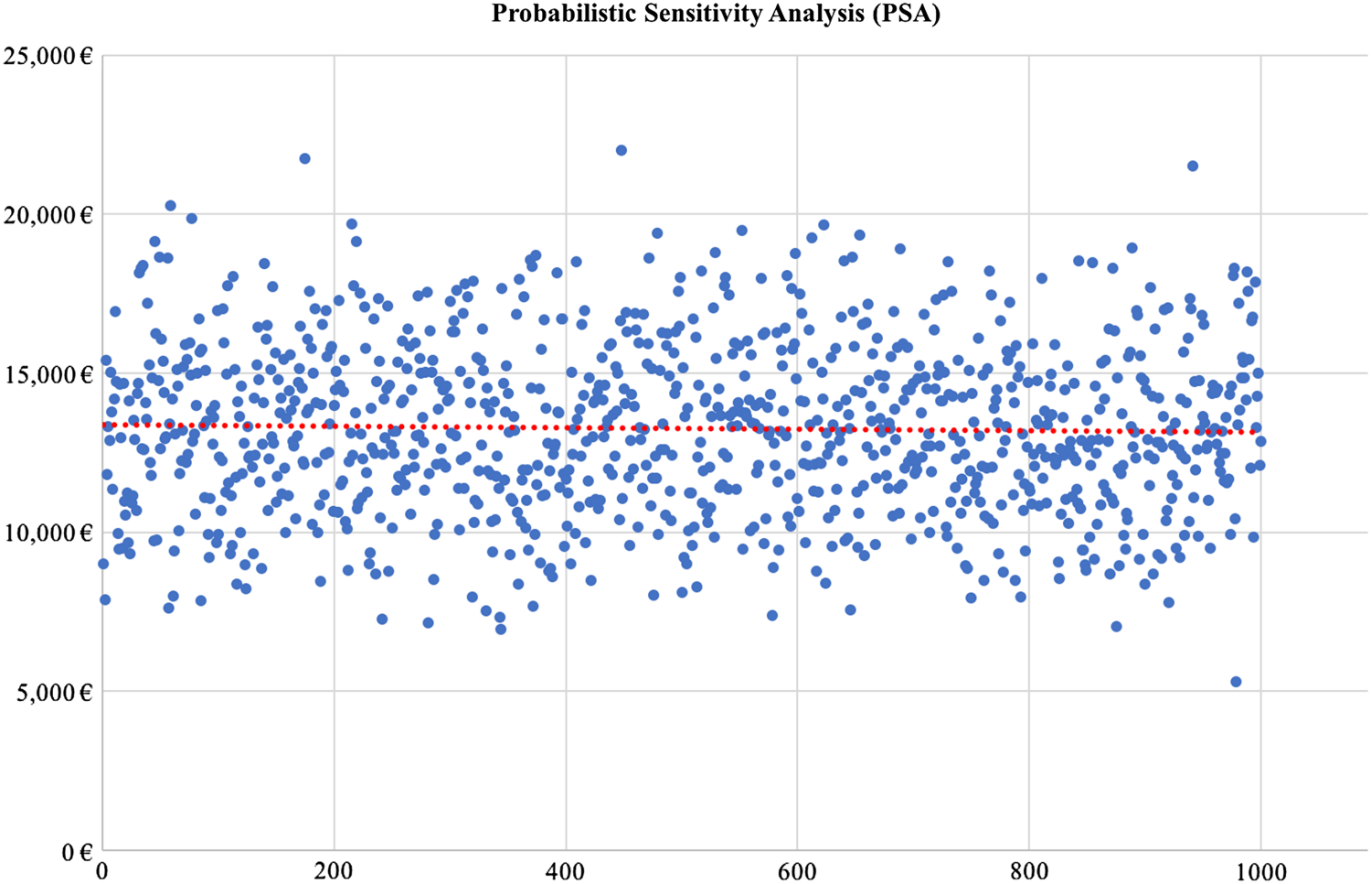
Probabilistic sensitivity analysis (PSA) performed to address uncertainty of parameter values when estimating the total burden of obesity. The PSA was performed adopting the Monte Carlo method (second order) and calculation of the total obesity costs was replicated with 1,000 simulations

In addition, the results were validated by performing a PSA (Fig. 2). After running 1,000 simulations of the model, the total costs of obesity displayed a 95% credible interval of €8.99 billion < µ < €17.80 billion.

## Discussion

This study aimed to estimate the direct and indirect costs attributable to obesity in Italy in 2020. The first challenge of this research was to choose reliable estimates of the obesity prevalence in the country. In Italy, there is currently no real consensus on the prevalence of obesity (BMI ≥ 30 kg/m^2^) which, according to the different sources, ranged between 10.8 [54] and 25.8% [18]. The most evident reason for this variation appeared to be the adopted data collection method. Studies that collected self-reported data reported on average ~12% less obese than the studies using measured data, thus underestimating the magnitude of the problem. This fact is supported by previous research confirming the underestimation of obesity prevalence when using self-reported data [40–42]. The present study used data from the Health Search database, which estimated an obesity prevalence of 22.2% (obesity class I: 72.8%; class II: 20.1%; class III: 7.2%) using measured data (Table 1) [53].

As the findings of the literature search did not show any Italian databases that provide data on obesity and contemporarily on all the obesity-associated pathologies included in the study, aggregate measures were retrieved from the literature (top–down approach). In addition, previous literature highlighted the advantage of adopting this approach when including multiple diseases in the economic evaluation. In fact, allocating the total country expenditures among the most important cost items minimises the risk that the sum of individual costs related to each included disease—estimated by adopting a bottom–up approach—is greater than the total national health care expenditure and thus minimises the risk of duplicating costs [76].

Direct costs were determined considering the costs of bariatric surgery and the costs of obesity-associated pathologies as main components of the total obesity burden. Previous research widely described the association of obesity with diabetes and CVDs, which were thus included in the analysis (Table 3). In addition, 11 types of cancer were considered due to the increasing evidence of their association with obesity [6, 47]. The contribution of cancer to the economic burden of obesity is currently scarcely explored in Italy and therefore represented a particularly interesting aspect for the present study. Direct costs amounted to €7.89 billion, the main drivers being CVDs (€6.66 billion) that accounted for ~ 49.9% of the overall costs, while the costs of bariatric surgery had the smallest impact on costs among the included cost items (€0.24 billion).

Costs of hypertension and diabetes were €0.84 and €0.65 billion, respectively (Table 6). These findings are in line with other studies that reported a similar impact of these two pathologies on the burden of obesity [29, 77]. Peculiar were the cases of ischaemia (€1.65 billion) and stroke (€1.72 billion), which doubled the costs of diabetes and hypertension (Table 6), likely due to differences in the cost selection performed prior to the cost evaluation. On one side, costs pro-capita of ischaemia were calculated as an average of the other CVDs and costs of stroke were taken from a study that did not account for the presence of comorbidities, thus probably resulting in an overestimation of costs [72]. On the contrary, diabetes and hypertension costs were obtained from studies that differentiated costs in the presence/absence of comorbidities [66, 69]. In this case, the lowest value was selected in a prudential mode to minimise duplication of costs with other pathologies, which likely lead to an underestimation of costs. In fact, the PAF implies a causal relationship with obesity and another pathology, thus already defining the patients as “comorbid”. In addition, obese and severely obese patients are more likely to present multiple comorbidities interacting together. A severely obese patient affected by diabetes and hypertension will then result in a greater need for healthcare resources compared to two patients independently affected either by diabetes or hypertension.

Costs attributable to bariatric surgery amounted to €0.24 billion, representing the expenses that result from the treatment of 1.4% of the eligible patient population [43]. This rate indicates a severe under-treatment of obese patients needing weight-loss surgeries in Italy. However, the 1.4% rate is in line with the ones reported for other countries in Europe, such as Germany (less than 1%) [51, 78], and UK (0.002%) [52]. Italy displayed a significantly lower rate of bariatric surgeries compared to France and Belgium, probably due to different accessibility criteria and financial rules [51]. In France in 2018, for example, more than 30% of surgeries were performed on patients with a BMI < 40 kg/m^2^ and mostly in the absence of comorbidities [79].

Costs associated with GP visits, counselling programmes and special transportation (i.e. bariatric ambulance, which requires a supplemental price compared to a regular ambulance) were excluded due to the lack of data in the literature. For example, previous studies did not differentiate between GP visits attributable to obesity alone and obesity-associated pathologies [74, 75]. The exclusion of these categories resulted in underestimating direct costs. However, these costs are negligible when compared with the economic impact of pathologies such as diabetes or CVDs.

Indirect costs amounted to €5.45 billion (€2.62 for absenteeism; €2.83 for presenteeism) (Table 7). Costs pro-capita were taken from Gupta et al., which estimated the indirect costs of obesity in five countries, including Italy, using the human capital approach [50]. However, Gupta et al. did not report costs at a population level and thus did not allow a comparison with the results presented here. A recent review reported obesity-attributable productivity losses ranging from $89 to $1586 for absenteeism and $11 to $4175 for presenteeism, in line with the costs used in this analysis (average of €467 for absenteeism and €598 for presenteeism) [80]. Three German COI studies reported similar findings with the ones presented here: Effertz et al. estimated $4.97 billion of obesity-attributable indirect costs in 2016 [81], Konnopka et al. estimated €5 billion attributable to both overweight and obesity in 2002 [77], and Knoll et al. projected €3.6 billion of obesity-attributable indirect costs in 2020 [82]. The costs reported by Effertz et al. [81] and Konnopka et al. [77] represent, respectively, ~ 1.4% and ~ 2.2% of the German health expenditure for the years of reference [83, 84], compared to the €5.5 billion reported in this study, which account for ~ 4.8% of the Italian health expenditure (considering the health expenditure of 2019) [85]. A COI study performed in Canada in 2006 by Klarenbach et al. estimated lower costs for absenteeism compared to this study ($187 million vs. €676 million for class III obese) [86]. However, Klarenbach et al. used self-reported data on BMI, which, as previously discussed, may have resulted in underestimating costs. Higher indirect costs were reported by studies performed in the USA, likely due to the differences in obesity prevalence compared to Italy. For example, Ricci and Chee estimated $11.7 billion, considering a 42% prevalence [87].

According to the findings reported here, the total burden of obesity in Italy for 2020 is €13.34 billion. These costs are higher than those proposed by Atella et al. in 2012, which estimated a burden of €4.5 billion adopting a population-based approach (bottom–up) and using prevalence data from Health Search [17, 29]. A reason for this discrepancy could be the top–down approach of this study and the use of the PAF, which may have resulted in a duplication of costs. The COI study by Konnopka et al. estimated total obesity- and overweight-attributable costs of ~ €10 billion in 2002 in Germany [77]. If adjusted to 2020 the overall costs amount to ~ €12 billion in line with the findings presented in this study.

The uncertainty of the data included in the model was addressed by performing a deterministic sensitivity analysis and a PSA (Figs. 1, 2 ). Varying the prevalence of obesity had the highest impact on the total obesity costs, determining a €2.4-billion reduction on overall obesity costs when the obesity prevalence was decreased by 10%. This result further underlined the key importance of accurate measures of prevalence in economic evaluations of obesity. Similarly, the costs of CVDs and diabetes had a major impact on overall obesity costs compared to the rate of bariatric surgery, the latter increasing the total costs of obesity by €350 million when doubled. The PSA returned a credible interval of €8.99 billion < µ < €17.80 billion.

A major challenge that occurred when comparing results from similar previously published COI studies was the heterogeneity of contextual factors across studies, such as drug pricing, demographic characteristics of the population, and healthcare system organisation [88, 89]. Accounting for different contextual factors is particularly important when adopting a healthcare system perspective, for example in the context of a public or private healthcare system. It is therefore important to have studies that specifically address the burden of obesity in Italy, to compare results from studies that have similar contextual factors and tackle the challenge of data transferability.

The COI model developed in this study presents valuable strengths. A validation tool was developed to select the most reliable and accurate source for data on prevalence (Supplementary Table 5), as Italy currently lacks a univocal agreement on prevalence of obesity in the country. Adopting a validation tool ensured the selection of a source that satisfied a number of selection criteria describing completeness, accuracy, comparability and clarity of data. Another strength of the study is represented by the fact that the contribution of each obesity-associated pathology was evaluated separately (Table 6), aiming to accurately portray the “weight” of each pathology. For example, Konnopka et al. grouped pathologies in categories (neoplasms, CVDs etc.) and assumed equal costs for diseases in the same category [77]. However, pathologies within a category may differ in costs, as for hypertension and stroke, which in the present study were associated with direct costs of €272 and €7419, respectively. In addition, this study provides concrete figures for the burden of obesity in Italy at a population level (Table 8), while previous studies only reported an increased percentage of healthcare utilisation or average costs pro-capita [29, 90]. Finally, the study results were validated by performing a deterministic sensitivity analysis and a PSA as discussed above.

This study presents limitations that need to be considered. First, children and adolescents were excluded from the analysis due to the heterogeneity of input data sources, thus underestimating the real burden of obesity in Italy. In addition, this study does not consider variations in direct costs between obesity classes (I, II, III). Therefore, this study does not account for the fact that severely obese patients (class III) have been reported to display an increased risk for several CVDs and multi-morbidity compared to class I and II patients and therefore have a greater impact on the overall burden of obesity [91]. Most of the RRs used for the PAFs were collected in the US, and transferring these risk measures to Italy may result in uncertainty (Tables 3 and 4). Moreover, this study does not consider that obese people often present multiple comorbidities that have a “synergistic” rather than “additive” effect and display specific dynamics that might not be reflected in the PAF. Another limitation of the PAF is its consideration of all patients affected by a certain pathology on the same level regardless of the exposure (e.g. obese or not obese). However, a normal-weight patient with a single pathology costs on average less than an obese patient with the same pathology. Choosing for example a cost pro-capita of €353 solely attributable to diabetes represents a highly prudential choice because an obese patient affected by diabetes holds a higher probability to be contemporary affected by a CVD compared to a normal-weight diabetic. The use of the PAF may also lead to accounting costs of the included pathologies more than once. For example, using the top–down approach to estimate the costs of the comorbidity “diabetes” and retrieving the value from another study would lead to accounting also for costs attributed to CVDs resulted from diabetes, which in our study are considered separately. To minimise the risk of double counting, we filtered out cost categories referring to comorbidities when extracting the costs pro-capita for each pathology from the literature. Finally, an average cost per person for all cancers was considered for the cost evaluation due to the lack of country-specific data in the literature. However, the assumption of an equal cost per person among different cancer types might have resulted in a simplification and consequent underestimation or overestimation of costs.

For future research, it is advisable to include other pathologies (e.g. dyslipidaemia and arthritis), and consider cost figures specific for each type of obesity-associated pathology included in the analysis (i.e. specific costs for each included cancer type). It is also advisable to include children and adolescents, excluded in this study due to the lack of suitable data for Italy, at present displaying a marked heterogeneity in collection methods, as well as in the definitions of age cutoffs and BMI classes. We obtained data for this study using a snow-ball method instead of a systematic review approach due to the fact that we started our search based on three key national reports that provided specific figures for Italy. A snow-ball method approach appeared more effective since it allowed us to start from a number of key references and gradually widen the scope.

In conclusion, this study represents the first comprehensive COI study of obesity in Italy which estimated direct healthcare costs of the most common obesity-associated pathologies and indirect costs due to productivity losses. The findings indicated high obesity-attributable costs, calling for action on developing cost-effective prevention programmes in Italy. This study also underlines the lack of reliable data on obesity prevalence for Italy, which constitutes the basis for sound economic evaluations. Further effort should be put in developing more reliable data collection to improve homogeneity and comparability of results and to reach a national consensus on the cost of obesity.

## Supplementary Information

Below is the link to the electronic supplementary material.

Supplementary file1 (DOCX 59 KB)

## Data Availability

All disaggregate measures are available upon request to the authors.
