# Supplementary material for: The economic burden of obesity in Italy: a cost-of-illness study
Source: Eur J Health Econ. 2021 Aug 4;23(2):177–92. doi: 10.1007/s10198-021-01358-1 (PMC8882110; doi:10.1007/s10198-021-01358-1)
Supplement: Supplementary file 1 — Supplementary file1 (DOCX 59 KB) [file 10198_2021_1358_MOESM1_ESM.docx]

# Supplementary material

| ***Supplementary Table 1****: Quality criteria for external data sources according to the Essnet Validat Foundation.* |
| --- |
| **Criteria as reported by the Essnet Validat Foundation [39]** |
| Completeness |
| Peer review |
| Formal methods |
| Redundancy |
| Accuracy |
| Coherence and comparability |
| Clarity and accessibility |
| Timeliness |

| ***Supplementary Table 2****: Main characteristics of the included databases.* | | | | | |
| --- | --- | --- | --- | --- | --- |
| **Source, year** | **Sample size (n)** | **Age range (years)** | **Adults defined as: (years)** | **Data collection** | **BMI classification** |
| Health Search, 2012 [29,53] | 557,145 | 18–65+ | ≥ 18 | measured | Underweight: BMI <18.5  Normal weight: BMI ≥ 18.5 to <25 Overweight: BMI ≥ 25 to <30 Obese: BMI ≥ 30 (I: ≥ 30 to <35; II: ≥ 35 to <40; III: ≥ 40) |
| Progetto CUORE, 2012 [18,44] | 8,141 | 35–74 | ≥ 18 | measured | Underweight: BMI < 18.5  Normal weight: BMI ≥ 18.5 to <25 Overweight: BMI ≥ 25 to <30  Obese: BMI ≥ 30 |
| Global Burden of Disease, 2015 [6,64] | multi | 0–80+ | > 20 | mixed (measured and  self-reported) | Overweight: BMI ≥ 25 to <30 Obese: BMI ≥ 30 |
| EUROSTAT, 2015 [54] | 21,776 | 15–75+ | >15 | self-reported | Underweight: BMI < 18.5  Normal weight: BMI ≥ 18.5 to <25 Overweight: BMI ≥ 25 to <30  Obese: BMI ≥ 30 |
| Global Health Observatory (WHO), 2016 [92] | not reported | > 5 | ≥20 | measured | Underweight: BMI < 18.5  Normal weight: BMI ≥ 18.5 to <25 Overweight: BMI ≥ 25 to <30  Obese: BMI ≥ 30 |
| ISTAT, 2018 [93] | 37,391 | 0–80+ | ≥ 18 | self-reported | Underweight: BMI < 18.5  Normal weight: BMI ≥ 18.5 to <25 Overweight: BMI ≥ 25 to <30  Obese: BMI ≥ 30 |
| Indagine Passi, 2018 [94] | 132,158 | 18–69 | ≥ 18 | self-reported | Underweight: BMI < 18.5  Normal weight: BMI ≥ 18.5 to <25 Overweight: BMI ≥ 25 to <30  Obese: BMI ≥ 30 |
| *ISTAT = Istituto Nazionale per la Statistica; EUROSTAT = European Statistics* | | | | | |

| ***Supplementary Table 3****: Prevalence data of body-mass index (BMI) classes (obesity, overweight, normal, underweight) for adults in Italy from seven different databases.* | | | | | | | | | | |
| --- | --- | --- | --- | --- | --- | --- | --- | --- | --- | --- |
| **Source** | **Updated to** | **Year** | **Public** | **Data Type** | **Sample Size** | **Sex** | **Underweight  BMI<18.50 (%)** | **Normal  18.50≥BMI>25 (%)** | **Overweight 25≥BMI>30 (%)** | **Obese  BMI > 30 (%)** |
| ISTAT multiscopo stand*[93] | 2018 | 2017 | yes | self-reported | 41,100 |  | - | - | 34.6 | 10.2 |
| ISTAT multiscopo stand*[93] | 2018 | 2017 | yes | self-reported | 41,100 | M |  |  |  | 11.5 |
| ISTAT multiscopo stand*[93] | 2018 | 2017 | yes | self-reported | 41,100 | F |  |  | 34.1 | 8.9 |
| ISTAT multiscopo†[93] | 2018 | 2017 | yes | self-reported | 41,100 |  | 3.01 | 51.03 | 35.37 | 10.59 |
| ISTAT multiscopo†[93] | 2020 | 2018 | yes | self-reported | 37,391 |  | 3 | 50.26 | 35.76 | 10.98 |
| EUROSTAT (EHIS) [54] | 2020 | 2015 | yes | self-reported | 21,776 | M/F | 3.3 | 51.9 | 34.1 | 10.8 |
| EUROSTAT (EHIS) [54] | 2020 | 2015 | yes | self-reported | 21,776 | M | 1.3 | 45.5 | 42.1 | 11 |
| EUROSTAT (EHIS) [54] | 2020 | 2015 | yes | self-reported | 21,776 | F | 5.8 | 58.9 | 25.3 | 10.1 |
| Health Search[29] | 2015 | 2010 | no | measured | 557,145 | M/F | 2.66 | 40.8 | 36.88 | 19.66 |
| Health Search^55^ | 2019 | 2014 | no | measured | 557,145 | M/F | - | - | - | - |
| Health Search[53] | 2017 | 2012 | no | measured | 20,159 | M/F | 2.3 | 38.1 | 37.4 | 22.2 |
| Global Burden of Disease**[6] | 2014 | 2013 | no | mixed | multi | M | - | - | 58.3 (95%CI: 55.5-61.1) | 18.6 (95%CI: 16.9-20.4) |
| Global Burden of Disease**[6] | 2014 | 2013 | no | mixed | multi | F | - | - | 41.4 (95%CI: 38.9-44.2) | 17.7 (95%CI: 15.9-19.5) |
| Global Burden of Disease**[64] | 2017 | 2015 | no | mixed | multi | M | - | - | 42.87 (95%CI: 41.3-44.62) | 10.86 (95%CI: 9.6-12.2) |
| Global Burden of Disease**[64] | 2017 | 2015 | no | mixed | multi | F | - | - | 25.14 (95%CI: 23.96-26.51) | 11.08 (95%CI: 9.85-12.37) |
| Global Health Observatory**[92] | 2020 | 2016 | no | measured | multi | M | 0.4 (95%CI: 0.2-0.7) |  | 65.3 (95%CI: 59.7-70.7) | 20.1(95%CI: 15.3-25.2) |
| Global Health Observatory**[92] | 2020 | 2016 | no | measured | multi | F | 1.8 (95%CI: 0.9-3) |  | 51.5 (95%CI: 45.6-57.6) | 19.5 (95%CI: 15.0-24.6) |
| Global Health Observatory**[92] | 2020 | 2016 | no | measured | multi | M/F | 1.1 (95%CI: 0.6-1.7) |  | 58.5(95%CI: 54.3-62.5) | 19.9 (95%CI: 16.6-23.4) |
| Global Health Observatory†[92] | 2020 | 2016 | no | measured | multi | M | 0.3 (95%CI: 0.1-0.5) |  | 70.1 (95%CI: 64.2-75.5) | 22.5 (95%CI: 17.4-27.9) |
| Global Health Observatory†[92] | 2020 | 2016 | no | measured | multi | F | 1.3 (95%CI: 0.7-2.3) |  | 58.6 (95%CI: 52.7-64.5) | 23.3 (95%CI: 18.2-28.9) |
| Global Health Observatory†[92] | 2020 | 2016 | no | measured | multi | M/F | 0.8 (95%CI: 0.5-1.3) |  | 64.1 (95%CI: 60.1-68.2) | 22.9 (95%CI: 19.3-26.8) |
| Indagine Passi[94] | 2020 | 2015/18 | no | self-reported | 132,158 | M | - | - | 39.3 (95%CI: 38.8 - 39.8) | 11.4 (95%CI: 11.1 - 11.8) |
| Indagine Passi[94] | 2020 | 2015/18 | no | self-reported | 132,158 | F | - | - | 24.0 (95%CI: 23.6 - 24.4) | 10.3 (95%CI: 10.0 - 10.6) |
| Indagine Passi[94] | 2020 | 2015/18 | no | self-reported | 132,158 | M/F | 57.6 (95%CI: 57.2 - 57.9) | | 31.6 (95%CI: 31.3 - 31.9) | 10.9 (95%CI: 10.6 - 11.1) |
| CUORE (OEC/HES)[18] | 2015 | 2008/12 | yes | measured | 8,141 | M | 0.2 | 27.8 | 47.4 | 24.6 |
| CUORE (OEC/HES)[18] | 2015 | 2008/12 | yes | measured | 8,141 | F | 1.4 | 41.8 | 32 | 24.8 |
| **Standardized estimates to the EU standard population; †Crude estimates; **Age–standardized estimates* | | | | | | | | | | |

***Supplementary Table 4****: Original definition of validation and quality criteria for external data sources according to the Essnet Validat Foundation and adoption in this study in the burden of obesity in Italy.*

| **Criteria as reported**  **by the Essnet Validat Foundation^35^** | **Adopted**  **in this study** | **Corresponding criteria in supplementary table 5** |
| --- | --- | --- |
| Completeness | ✓ | 3, 4 |
| Peer review | ✓ | 1, 8, 9 |
| Formal methods | ✓ | 5, 6 |
| Redundancy |  |  |
| Accuracy | ✓ | 2, 3, 4 |
| Coherence and comparability | ✓ | 7 |
| Clarity and accessibility | ✓ | 6 |
| Timeliness |  |  |

***Supplementary Table*** ***5****: Criteria for the validation of the secondary source for the prevalence input data used to estimate the economic burden of obesity in Italy*

|  |  | Health Search[29,53] | CUORE[18,44] | GBD[6,64] | EUROSTAT[54] | GHO(WHO)[92] | ISTAT[93] | PASSI[94] |
| --- | --- | --- | --- | --- | --- | --- | --- | --- |
| 1. | **Are the data from a reliable source (e.g. government, international accredited institution, research paper)?** Yes = 1 point; No = 0 points | 1 | 1 | 1 | 1 | 1 | 1 | 1 |
| 2. | **Which data type has been collected?** Measured = 2 points; Mixed = 1 point; Self-reported = 0 points | 2 | 2 | 1 | 0 | 2 | 0 | 0 |
| 3. | **Does the data source cover the time frame, geographical area, and variable that is relevant to the study?** Yes = 1 point; No = 0 points | 1 | 1 | 1 | 1 | 1 | 1 | 0 |
| 4. | **Does the database provide measurements for all BMI classes?**  Yes = 1 point; No = 0 points | 1 | 0 | 0 | 1 | 1 | 1 | 1 |
| 5. | **Is the sample size for Italy reported?** Yes = 1 point; No = 0 points | 1 | 1 | 0 | 1 | 0 | 1 | 1 |
| 6. | **Is the data collection methodology clearly explained?** Yes = 1 point; No = 0 points | 1 | 1 | 1 | 1 | 1 | 1 | 1 |
| 7. | **Are the database measurements consistent with other sources?**  Yes = 1 point; No = 0 points | 1 | 0 | 1 | 0 | 1 | 0 | 1 |
| 8. | **Have been the measurements of the database used in previous research?** Yes = 1 point; No = 0 points | 1 | 1 | 1 | 1 | 1 | 1 | 1 |
| 9. | **Has been the database validity confirmed by a field expert?** Yes = 1 point; No = 0 points | 1 | 1 | 1 | 1 | 1 | 1 | 1 |
|  | **Total points** | 10 | 8 | 7 | 7 | 9 | 7 | 7 |

| ***Supplementary Table 6****: Data on prevalence of* *cardiovascular diseases (CVDs) and diabetes in the Italian adult population.* | | | | |
| --- | --- | --- | --- | --- |
| **Disease** | **Age** | **Year** | **Prevalence (Pe) %** | **Reference** |
| Angina | > 35 | 2014 | 0.8 | Atella et al., 2019^55^ |
| Atrial fibrillation | > 35 | 2014 | 2.9 | Atella et al., 2019^55^ |
| Cerebrovascular disease | 35–74 | 2014 | 0.70 | Giampaoli et al., 2015^39^ |
| Diabetes | > 35 | 2014 | 8 | Atella et al., 2019^55^ |
| Heart Failure | > 35 | 2014 | 1.3 | Atella et al., 2019^55^ |
| Hypertension* | > 35 | 2014 | 30 | Atella et al., 2019^55^ |
| Myocardial infarction | 35-74 | 2014 | 1.00 | Giampaoli et al., 2015^39^ |
| Other Ischemia (Acute and Chronic) | > 35 | 2014 | 3.50 | Atella et al., 2019^55^ |
| Pulmonary Embolism | - | 2007 | 0.0189 | Moretti et al., 2010^56^ |
| Stroke | > 35 | 2014 | 4.9 | Atella et al., 2019^55^ |
| Vascular Disease | > 35 | 2014 | 3.1 | Atella et al., 2019^55^ |

***Supplementary Table 7****: Calculation of the Population Attributable Fraction (PAF) of comorbidities associated with obesity, using relative risks (RRs) extracted from four different studies.*

| **Obesity-associated pathology** | **RR*** | **RR-1** | **Prevalence (Pe)  of obesity** | **Pe(RR-1)** | **PAF** | **Reference** |
| --- | --- | --- | --- | --- | --- | --- |
| Angina | 1.96 | 0.96 | 0.222 | 0.213 | 18% | Wilson et al., 2002 |
| Atrial fibrillation | 1.49 | 0.49 | 0.222 | 0.109 | 10% | Wanahita et al., 2008 |
| Breast cancer | 1.25 | 0.25 | 0.222 | 0.056 | 5% | DYNAMO-HIA, 2010 |
| Cerebrovascular disease | 1.54 | 0.54 | 0.222 | 0.120 | 11% | Wilson et al., 2002 |
| Colorectal cancer | 1.25 | 0.25 | 0.222 | 0.056 | 5% | DYNAMO-HIA, 2010 |
| Congestive heart failure | 1.79 | 0.79 | 0.222 | 0.175 | 15% | Guh et al., 2009 |
| Diabetes | 6.25 | 5.25 | 0.222 | 1.166 | 54% | DYNAMO-HIA, 2010 |
| Hypertension | 2.41 | 1.41 | 0.222 | 0.313 | 24% | Global Burden of Disease, 2015 |
| Ischemia | 2.00 | 1.00 | 0.222 | 0.222 | 18% | DYNAMO-HIA, 2010 |
| Kidney cancer | 1.68 | 0.68 | 0.222 | 0.151 | 13% | DYNAMO-HIA, 2010 |
| Leukaemia | 1.11 | 0.11 | 0.222 | 0.024 | 2% | Global Burden of Disease, 2015 |
| Liver cancer | 1.24 | 0.24 | 0.222 | 0.053 | 5% | Global Burden of Disease, 2015 |
| Myocardial infarction | 1.44 | 0.44 | 0.222 | 0.098 | 9% | Wilson et al., 2002 |
| Oesophageal cancer | 2.30 | 1.30 | 0.222 | 0.289 | 22% | DYNAMO-HIA, 2010 |
| Ovarian cancer | 1.04 | 0.04 | 0.222 | 0.009 | 1% | Global Burden of Disease, 2015 |
| Pancreatic cancer | 1.08 | 0.08 | 0.222 | 0.018 | 2% | Global Burden of Disease, 2015 |
| Prostate cancer | 1.05 | 0.05 | 0.222 | 0.011 | 1% | Guh et al., 2009 |
| Pulmonary embolism | 3.51 | 2.51 | 0.222 | 0.557 | 36% | Guh et al., 2009 |
| Stroke | 1.56 | 0.56 | 0.222 | 0.124 | 11% | DYNAMO-HIA, 2010 |
| Thyroid cancer | 1.18 | 0.18 | 0.222 | 0.040 | 4% | Global Burden of Disease, 2015 |
| Uterine cancer | 1.61 | 0.61 | 0.222 | 0.135 | 12% | Global Burden of Disease, 2015 |
| **average of RR reported for female and male* | | |  |  |  |  |
